# Security Threat Assessment of an Internet Security System Using Attack Tree and Vague Sets

**DOI:** 10.1155/2014/506714

**Published:** 2014-10-21

**Authors:** Kuei-Hu Chang

**Affiliations:** Department of Management Sciences, R.O.C. Military Academy, Kaohsiung 830, Taiwan

## Abstract

Security threat assessment of the Internet security system has become a greater concern in recent years because of the progress and diversification of information technology. Traditionally, the failure probabilities of bottom events of an Internet security system are treated as exact values when the failure probability of the entire system is estimated. However, security threat assessment when the malfunction data of the system's elementary event are incomplete—the traditional approach for calculating reliability—is no longer applicable. Moreover, it does not consider the failure probability of the bottom events suffered in the attack, which may bias conclusions. In order to effectively solve the problem above, this paper proposes a novel technique, integrating attack tree and vague sets for security threat assessment. For verification of the proposed approach, a numerical example of an Internet security system security threat assessment is adopted in this paper. The result of the proposed method is compared with the listing approaches of security threat assessment methods.

## 1. Introduction

Due to information age's advance, security threat assessment of an Internet security system has become much more important and complicated. To ensure information security, many organizations use firewalls to provide a level of security by controlling access to information systems. A security manager has to make a decision and choose to implement a subset of these policies in order to maximize resource utilization. There has now been extensive research on security threat assessments; for some recent examples, see Tidwell et al. [1], Dhillon and Torkzadeh [2], Satoh et al. [3], Symantec Corporation [4], Opdahl and Sindre [5], Wu and Ye [6], Lee and Chang [7], and Blyth [8]. Helmer et al. [9] proposed the Multi-Agents Intrusion Detection System (MAIDS), which uses mobile agents in a distributed system to obtain audit data, correlate events, and discover intrusions. They used software fault trees to define intrusions and develop the requirement model for intrusion detection systems. Azaiez and Bier [10] used optimal attack strategies by analogy with existing results for the least expected cost failure state diagnosis of reliability systems. In addition, the growing popularity of e-government services on the Internet has also brought with it security threats. Similarly, J. J. Zhao and S. Y. Zhao [11] assessed the security of US state e-government sites to identify opportunities for and threats to the sites and their users. They used a combination of three methods—web content analysis, information security auditing, and computer network security mapping—for data collection and analysis.

**Figure 1:**
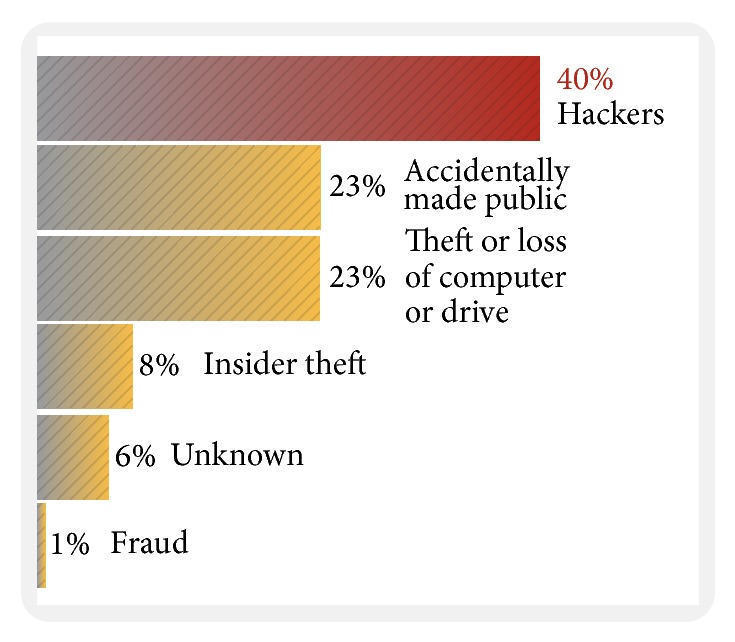
Top causes of data breaches [4].

The increasing frequency and complexity of Internet attacks have raised the level of sophistication required by systems administrators to effectively cope with script kiddies and more sophisticated hackers, for example, top causes of data breaches of Symantec Corporation in 2012, as shown in Figure 1. Hackers continue to be responsible for the largest number of data breaches, making up 40 percent of all breaches [4]. A secure computer system provides guarantees regarding the confidentiality, integrity, and availability of its data. However, systems generally contain design and implementation flaws that result in security vulnerabilities [9]. In addition, due to uncertainties and imprecision of data, it may be difficult or even impossible to precisely determine the failure probabilities of components. On the other hand, the incomplete failure data of the bottom events suffered in the attack also increase the difficulty of security threat assessment and calculation. It cannot be fully solved by traditional probability reliability. Therefore, this study used a vague set approach to overcome this problem. The concept of vague set was proposed by Gau and Buehrer [12]. A great deal of literature [13–19] has been carried out in vague set methods.

An attack tree supports design and required decisions. Attack trees are thus a formalized and structured method for analyzing threats. The possible decomposition of an attack tree to divide the goal into subgoals is an interesting alternative to explore. It is also known as a fault tree [20]. In 1999, Schneier was the first to propose attack trees to analyze the security of systems and subsystems [20]. Attacks are represented in a tree structure, with the attacker goal as the root node and the different ways of achieving that goal as leaf nodes. The attack tree includes the “AND” node and the “OR” node. To reach an AND node, all subgoals must be achieved. To reach an OR node, at least one of the subgoals must be achieved. The attack tree is a formal and methodical way of describing the security of the system based on varying attacks.

In reliability assessment, when the malfunction data of the system's elementary event are incomplete, the conventional approach of calculating reliability is no longer applicable [21]. Huang et al. [22] proposed the posbist fault tree analysis method to find a system's reliability by redefining the “AND” and “OR” operators based on the minimal cut of a posbist fault tree. However, their method only selects the maximal failure probability of the bottom event, which can result in biased conclusions. To solve this problem, this paper proposes a novel security threat assessment method that collects experts' knowledge and experience on the problem domain to build the possibility of the failure of leaf nodes through integrating attack tree and vague sets to assess security threats of an Internet security system. A security threat assessment of an Internet security system is presented as a case study to further illustrate the proposed method. It also compares the proposed approaches with several other listed methods in this paper.

The rest of this paper is organized as follows.  Section 2 introduces the basic definition and operations of the attack tree.  Section 3 introduces the basic definition and operations of the vague sets.  Section 4 presents the proposed approach, which integrates the attack tree and the vague sets for safety assessment. A numerical example of an Internet security system is adopted, and some comparisons with the listed approaches are discussed in Section 5. The final section makes conclusions.

## 2. Attack Tree

Schneier [20] proposed attack trees to analyze the security of systems and subsystems. It is a catalog of all possible attacks against a system. The purpose of the attack tree is to define and analyze possible attacks on a system in a structured way. The attack trees provide a formal, methodical way of describing the security of systems, based on varying attacks. An attack tree is initiated by a root node describing a type of attack, and each path is terminated by a leaf node (no children). Nodes can be decomposed by “AND” and “OR” relations.

**Figure 2:**
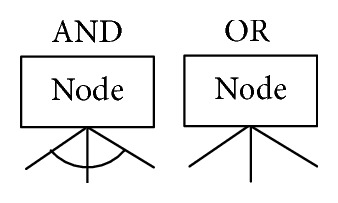
“AND” node and “OR” node.

If we let *x*_*j*_ be a random variable such that *x*_*j*_ = 1 corresponds to the accomplishments of subtask *j* and *x*_*j*_ = 0 corresponds to the failure of task *j*, then *P*(*x*_1_, *x*_2_,…, *x*_*n*_) is the joint probability distribution. In an “AND” node (see Figure 2), it must have *P*(*x*_1_ = 1, *x*_2_ = 1,…, *x*_*n*_ = 1). The accomplishment for the parent goal requires the success of all children—that is, *P*_and_ = ∏_*j*=1_^*n*^ *p*_*j*_—which is the product of the probability of accomplishments of all children. In an “OR” node (see Figure 2), this is essentially the negation of the probability that all subtasks fail: 1 − *P*(*x*_1_ = 0, *x*_2_ = 0,…, *x*_*n*_ = 0). The accomplishment for the parent goal requires the success of any one of the children—that is, *P*_or_ = 1 − ∏_*j*=1_^*n*^(1 − *p*_*j*_)—which is the product of the probability of an accomplishment of any one of the children. It assumes that the attacker can try all available subtasks until he finds one that succeeds. This is an unrealistic assumption in attack modeling, because if an attacker needs to try more than one subtask, he has manifested at least one failure. This is a situation that may be untenable in an attack. Therefore, Yager [23] assumed that in an “OR” node, where the attacker needs only to succeed at one subtask, he cannot try all possibilities but must try one. Thus, the probability of success at an “OR” node without any failure is *P*_OR_ = Max_*j*_[*P*_*j*_]. It is also clear that *P*_OR_ = Max_*j*_[*P*_*j*_] ≥ *P*_AND_ = ∏_*j*=1_^*n*^ *p*_*j*_.

## 3. Vague Sets and Their Operations

This section introduces the definitions and properties of vague sets and four arithmetic operations of the triangle vague set.

### 3.1. Definitions and Properties of Vague Sets [24]

Zadeh [25] proposed fuzzy sets to describe fuzzy phenomena under a specific attribute. A fuzzy set *F* is a class of objects, along with a grade of membership function. This membership function, *μ*_*F*_(*x*), *x* ∈ *X*, assigns a grade membership to each object that ranges between 0 and 1. This single value combines the evidence for *x* ∈ *X* and the evidence against *x* ∈ *X*, without indicating how much there is of each value. The notion of an intuitionistic fuzzy set was introduced for the first time by Atanassov [26] in 1983 as a generalization of an ordinary Zadeh fuzzy set. Let a set *X* be fixed. An intuitionistic fuzzy set *A* in *X* is an object that has the form *A* = {〈*x*, *μ*_*A*_(*x*), *ν*_*A*_(*x*)∣*x* ∈ *X*〉}, where the functions *μ*_*A*_(*x*) : *X* → [0,1] and *ν*_*A*_(*x*) : *X* → [0,1] define the degree of membership and the degree of nonmembership of the element *x* ∈ *X* to the set *A*; moreover, 0 ≤ *μ*_*A*_(*x*) + *v*_*A*_(*x*) ≤ 1 must hold.

**Figure 3:**
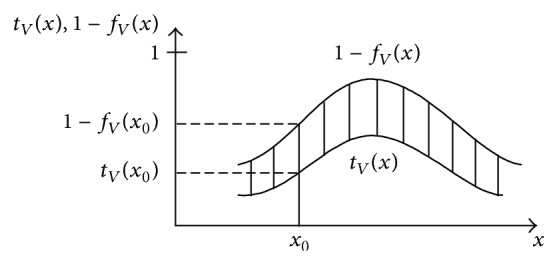
Vague set explanation of a real number *R*.

The concept of the vague set was proposed by Gau and Buehrer [12]. In a vague set *V*, for assigning a membership grade to every phenomenon, this membership grade is an interval of [0, 1]. This interval presents accepted evidence of *x* ∈ *X* and declined evidence at the same time. In membership grade *μ*_*V*_(*x*), a vague set *V* uses a truth-membership function *t*_*V*_ and a false-membership function *f*_*v*_ to represent the lower bound (*t*_*V*_) and upper bound (1 − *f*_*v*_). The interval [*t*_*V*_(*x*), 1 − *f*_*V*_(*x*)] can extend the fuzzy set of the membership function. In 1996, Bustince and Burillo [27] proposed that vague sets are intuitionistic fuzzy sets. The membership grade *μ*_*V*_(*x*) is not clear, but it is located in the subinterval [*t*_*V*_(*x*), 1 − *f*_*V*_(*x*)]  (i.e., *t*_*V*_(*x*) ≤ *μ*_*V*_(*x*) ≤ 1 − *f*_*V*_(*x*)) and 0 ≤ *t*_*V*_(*x*) + *f*_*V*_(*x*) ≤ 1. For example, if [*t*_*V*_(*x*_*i*_), 1 − *f*_*V*_(*x*_*i*_)] = [0.6,0.9], then *t*_*V*_(*x*_*i*_) = 0.6, 1 − *f*_*V*_(*x*_*i*_) = 0.9, *f*_*V*_(*x*_*i*_) = 0.1. The result can explain that *x*_*i*_ belongs to vague set *V* and accepts that the evidence is 0.6 and the declined evidence is 0.1. If *x*_*i*_ is the vote result from 10 people, it implies that six people voted in favor, one person voted against, and three persons abstained.  Figure 3 shows a vague set explanation of a real number *R*.

The uncertainty of *x* can be described as the differential value of (1 − *f*_*V*_(*x*)) − *t*_*V*_(*x*). If the differential value is small, it means that the value of *x* is more certain. If the differential value is great, it means that the computation is more uncertain about *x*. When 1 − *f*_*V*_(*x*) = *t*_*V*_(*x*), the vague set *V* regresses to a fuzzy set. Obviously, when 1 − *f*_*V*_(*x*) = *t*_*V*_(*x*) = 1 or 1 − *f*_*V*_(*x*) = *t*_*V*_(*x*) = 0, the vague set *V* regresses to a crisp set. From the above result, crisp sets and fuzzy sets can be viewed as special cases of vague sets. Therefore, vague sets can be used to describe vague objects in our daily life in more detail.

### 3.2. Arithmetic Operations of Triangle Vague Sets

**Figure 4:**
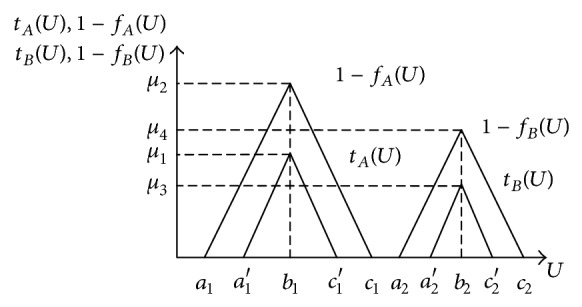
Triangle vague sets *A* and *B*.

Let *A* and *B* be two vague sets, as shown in Figure 4. If *t*_*A*_ ≠ *t*_*B*_ and *f*_*A*_ ≠ *f*_*B*_, then the arithmetic operations are defined as

(1) A=〈[(a1′,b1,c1′);μ1],[(a1,b1,c1);μ2]〉,B=〈[(a2′,b2,c2′);μ3],[(a2,b2,c2);μ4]〉,A(+)B=〈[(a1′,b1,c1′);μ1],[(a1,b1,c1);μ2]〉 (+)〈[(a2′,b2,c2′);μ3],[(a2,b2,c2);μ4]〉=〈[(a1′+a2′,b1+b2,c1′+c2′);min⁡(μ1,μ3)],  [(a1+a2,b1+b2,c1+c2);min⁡⁡(μ2,μ4)]〉,A(−)B=〈[(a1′,b1,c1′);μ1],[(a1,b1,c1);μ2]〉 (−)〈[(a2′,b2,c2′);μ3],[(a2,b2,c2);μ4]〉=〈[(a1′−c2′,b1−b2,c1′−a2′);min⁡⁡(μ1,μ3)],  [(a1−c2,b1−b2,c1−a2);min⁡(μ2,μ4)]〉,A(×)B=〈[(a1′,b1,c1′);μ1],[(a1,b1,c1);μ2]〉 (×)〈[(a2′,b2,c2′);μ3],[(a2,b2,c2);μ4]〉=〈[(a1′a2′,b1b2,c1′c2′);min⁡(μ1,μ3)],  [(a1a2,b1b2,c1c2);min⁡⁡(μ2,μ4)]〉,A(/)B=〈[(a1′,b1,c1′);μ1],[(a1,b1,c1);μ2]〉 (/)〈[(a2′,b2,c2′);μ3],[(a2,b2,c2);μ4]〉=〈[(a1′c2′,b1b2,c1′a2′)min⁡(μ1,μ3)],  [(a1c2,b1b2,c1a2);min⁡(μ2,μ4)]〉.

When *a*_1_ = *a*_1_′, *c*_1_ = *c*_1_′ and *a*_2_ = *a*_2_′, *c*_2_ = *c*_2_′, the vague sets of its four arithmetic operations will be easier.

## 4. Proposed Combination of an Attack Tree and Vague Sets Approach

### 4.1. The Reason for Using Attack Tree and Vague Sets

Security threat assessment of the Internet security system has become a greater concern in recent years, due to progress and diversification of information technology. For an Internet security system, due to uncertainties and imprecision of data, it may be difficult or even impossible to precisely determine the failure probabilities of components. Moreover, we must consider the failure probability of the bottom events suffered in the attack when security threat assessment is executed. Therefore, it cannot be fully solved by traditional probability reliability. An attack tree provides a way of modeling goals of an attack and alternative ways to achieve that goal. This helps us to study the system from the attackers' points of view, which may lead us to determine possible ways that the system can be compromised. Therefore, using an attack tree and vague sets approach to solve security threat assessment problems is more suitable. The major advantage of the vague set over the fuzzy set is that the vague set separates the positive (the degree of membership) and negative (the degree of nonmembership) evidence of membership of an element in the set. Fuzzy sets are vague sets, but the converse is not necessarily true. For this reason, it is more suitable to use the vague set, not the fuzzy set, in attack tree diagrams.

### 4.2. The Procedure of the Proposed Approach

According to the definitions in Section 3, this paper proposes six steps in order to implement vague attack tree analysis in security threat assessment of an Internet security system. The six steps are described as follows.

**Step 1 (construct the attack tree diagram).**

Construct the attack tree diagram by the AND node and OR node, tracing back the whole process from the main goal to the physical tasks.

**Step 2 (establish a system of reliability block diagram).**

A reliability block diagram can explain the units' relationships in parallel and in series.

**Step 3 (define the vague membership degree of leaf nodes).**

A unit fault can cause the breakdown of the whole system. Define the vague membership degree of leaf nodes according to an expert's knowledge and experience. Possible failure intervals of bottom events are obtained by aggregating group decision-making opinions of the experts' opinions.

**Step 4 (calculate the possible malfunction probability of the main goal).**

Use the attack tree diagram and vague set arithmetic operations to calculate the possible malfunction probability of the main goal.

**Step 5 (calculate the reliability of the main goal).**

The reliability of the main goal is equal to one minus the possible malfunction probability of the main goal.

**Step 6 (analyze the results and provide suggestions).**

From Step 5, the results can be further analyzed to provide the decision maker with feasible solutions.

## 5. An Illustrative Example

**Figure 5:**
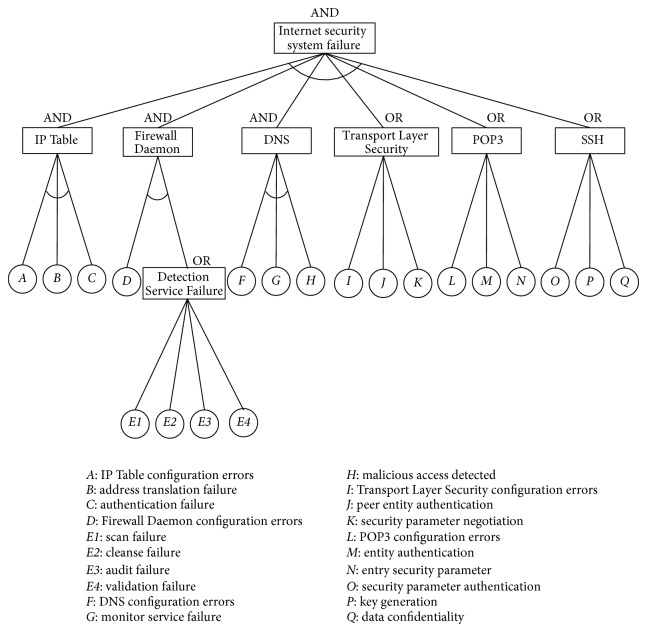
Attack tree of the Internet security system.

**Figure 6:**
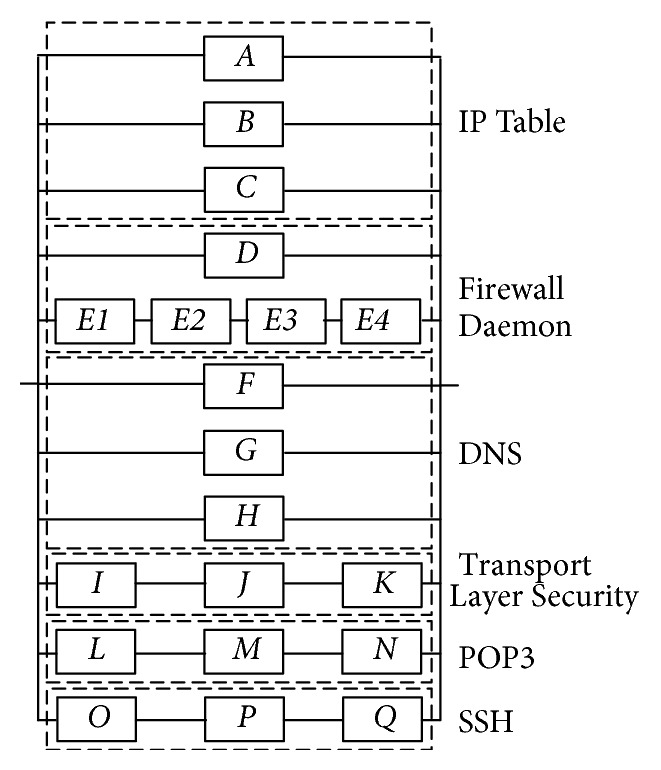
Parallel and series relationship of an attack tree diagram of the Internet security system.

In this section, an illustrative example of Internet security system failure during attack is presented in order to demonstrate the procedure that is proposed in this paper. This research also compares the experimental results with the traditional probability reliability and Huang et al.'s [22] methods. First of all, an attack tree is constructed that includes the main goal (the failure of the Internet security system during attack), the sub-goals (IP table, firewall daemon, domain name system (DNS), transport layer security, post office protocol 3 (POP3), secure shell (SSH)), the subtasks (Detection Service Failure), and the physical tasks (IP Table configuration errors, address translation failure, authentication failure, etc.). An attack tree integrates the main goal, the subgoals, the subtasks, and the physical tasks with “AND” and “OR” nodes (Figure 5). The descriptions of the physical tasks are listed in Table 1. Because of the incomplete failure data of system physical tasks, this paper proposes speculation by experts' opinions according to the incomplete information condition. The reliability block diagram of the Internet security system failure during attack is shown in Figure 6.

### 5.1. Traditional Probability Reliability

This research calculated the failure possibility of an “Internet security system” during attack, based on the data of Table 1 (column *b*_*i*_), by the traditional probability reliability method as follows:

(2) qT={1−(1−qA)(1−qB)(1−qC)(1−qD)  ×(1−qE1qE2qE3qE4)×(1−qF)(1−qG)(1−qH)  ×(1−qIqJqK)×(1−qLqMqN)×(1−qOqPqQ)}={1−(1−0.35)(1−0.15)(1−0.10)(1−0.05)  ×(1−0.13×0.11×0.05×0.04)  ×(1−0.10)(1−0.05)(1−0.40)  ×(1−0.14×0.20×0.30)×(1−0.18×0.25×0.40)  ×(1−0.09×0.07×0.04)}=1−0.2359=0.7641.

After the calculation above, it is shown that the failure probability of the “Internet security system” during attack is 0.2359 and the reliability of the “Internet security system” is 0.7641.

### 5.2. Huang et al. Method [22]

When the failure probability of a system is extremely small or when essential statistical data are scarce, the posbist fault-tree analysis proposed by Huang et al. [22] could be applied to predict and diagnose a system's failures and evaluate its reliability and safety. Calculations of the failure possibility of the “Internet security system,” based on the crisp failure possibilities, are listed in Table 1 (column *b*_*i*_), as per the following:

(3) Poss(SSH)=min⁡(Poss(O),Poss(P),Poss(Q))         =min⁡(0.09,0.07,0.04)=0.04,Poss(POP3)=min⁡(Poss(L),Poss(M),Poss(N))        =min⁡(0.18,0.25,0.40)=0.18,Poss(Transport  Layer  Security) =min⁡⁡(Poss(I),Poss(J),Poss(K)) =min⁡(0.14,0.20,0.30)=0.14,Poss(DNS)=max⁡(Poss(F),Poss(G),Poss(H))      =max⁡(0.10,0.05,0.40)=0.40,Poss(Detection  Service  Failure) =min⁡(Poss(E1),Poss(E2),Poss(E3),Poss(E4)) =min⁡(0.13,0.11,0.05,0.04)=0.04,Poss(Firewall  Daemon) =max⁡(Poss(D),Poss(Detection  Service  Failure)) =max⁡(0.05,0.04)=0.05,Poss(IP  Table)=max⁡(Poss(A),Poss(B),Poss(C))        =max⁡(0.35,0.15,0.10)=0.35.

Then, the top event possibilities of “Internet security system failure” during attacking can be calculated as

(4) Poss(Internet  Security  System  Failure) =max⁡⁡(Poss(SSH),Poss(POP3),      Poss(Transport  Layer  Security),Poss(DNS),      Poss(Firewall  Daemon),Poss(IP  Table)) =max⁡(0.04,0.18,0.14,0.40,0.05,0.35)=0.40.

After the calculation above, it is shown that the failure probability of the “Internet security system” during attack is 0.40 and the reliability of the “Internet security system” is 0.60.

### 5.3. Proposed Method

According to (1), the failure range of subgoals (SSH, POP3, Transport Layer Security, DNS, Detection Service Failure, Firewall Daemon, and IP Table) can be calculated as

(5) Pand(SSH) =PO×PP×PQ =〈[(0.08,0.09,0.10);0.8],[(0.07,0.09,0.11);1.0]〉  (×)〈[(0.06,0.07,0.08);0.8],[(0.05,0.07,0.09);0.9]〉  (×)〈[(0.03,0.04,0.05);0.9],[(0.03,0.04,0.05);0.9]〉 =〈[(0.0048,0.0063,0.0080);0.8],   [(0.0035,0.0063,0.0099);0.9]〉  (×)〈[(0.03,0.04,0.05);0.9],[(0.03,0.04,0.05);0.9]〉 =〈[(0.000144,0.000252,0.000400);0.8],   [(0.000105,0.000252,0.000495);0.9]〉,Pand(POP3) =PL×PM×PN =〈[(0.17,0.18,0.19);0.8],[(0.16,0.18,0.20);0.9]〉  (×)〈[(0.24,0.25,0.26);0.8],[(0.22,0.25,0.28);1.0]〉  (×)〈[(0.39,0.40,0.41);0.8],[(0.38,0.40,0.42);0.9]〉 =〈[(0.0408,0.0450,0.0494);0.8],   [(0.0352,0.0450,0.0560);0.9]〉  (×)〈[(0.39,0.40,0.41);0.8],[(0.38,0.40,0.42);0.9]〉 =〈[(0.015912,0.018000,0.020254);0.8],   [(0.013376,0.018000,0.023520);0.9]〉,Pand(Transport  Layer  Security) =PI×PJ×PK =〈[(0.13,0.14,0.15);0.8],[(0.12,0.14,0.16);0.9]〉  (×)〈[(0.16,0.20,0.24);0.8],[(0.15,0.20,0.25);1.0]〉  (×)〈[(0.28,0.30,0.32);0.8],[(0.27,0.30,0.33);0.9]〉 =〈[(0.0208,0.0280,0.0360);0.8],   [(0.0180,0.0280,0.0400);0.9]〉  (×)〈[(0.28,0.30,0.32);0.8],[(0.27,0.30,0.33);0.9]〉 =〈[(0.005824,0.008400,0.011520);0.8],   [(0.004860,0.008400,0.013200);0.9]〉,Por(DNS) =max⁡(PF,PG,PH) =max⁡{〈[(0.09,0.10,0.11);0.8],[(0.08,0.10,0.12);0.9]〉,       〈[(0.04,0.05,0.06);0.9],[(0.04,0.05,0.06);1.0]〉,      〈[(0.37,0.40,0.43);0.8],[(0.35,0.40,0.45);1.0]〉} =〈[(0.37,0.40,0.43);0.8],[(0.35,0.40,0.45);0.9]〉Pand(Detection  Service  Failure) =PE1×PE2×PE3×PE4 =〈[(0.12,0.13,0.14);0.9],[(0.11,0.13,0.15);1.0]〉  (×)〈[(0.10,0.11,0.12);0.9],[(0.10,0.11,0.12);1.0]〉  (×)〈[(0.04,0.05,0.06);0.9],[(0.03,0.05,0.07);1.0]〉  (×)〈[(0.03,0.04,0.05);0.9],[(0.03,0.04,0.05);1.0]〉 =〈[(0.0000144,0.0000286,0.0000504);0.9],   [(0.0000099,0.0000286,0.0000630);1.0]〉,Por(Firewall  Daemon) =max⁡(PD,PDetection  Service  Failure) =max⁡⁡{〈[(0.04,0.05,0.06);0.8],[(0.04,0.05,0.06);0.9]〉,       〈[(0.0000144,0.0000286,0.0000504);0.9],       [(0.0000099,0.0000286,0.0000630);1.0]〉} =〈[(0.04,0.05,0.06);0.8],[(0.04,0.05,0.06);0.9]〉,Por(IP  Table) =max⁡(PA,PB,PC) =max⁡{〈[(0.33,0.35,0.37);0.8],[(0.30,0.35,0.40);0.9]〉,       〈[(0.12,0.15,0.18);0.9],[(0.10,0.15,0.20);1.0]〉,       〈[(0.09,0.10,0.11);0.9],[(0.08,0.10,0.12);0.9]〉} =〈[(0.33,0.35,0.37);0.8],[(0.30,0.35,0.40);0.9]〉.

Then, the top event possibilities of “Internet security system failure” during attack can be calculated as

(6) Por(Internet  Security  System  Failure) =max⁡(PSSH,PPOP3,PTransport  Layer  Security,      PDNS,PFirewall  Daemon,PIP  Table) =max⁡{〈[(0.000144,0.000252,0.000400);0.8],       [(0.000105,0.000252,0.000495);0.9]〉,     〈[(0.015912,0.018000,0.020254);0.8],      [(0.013376,0.018000,0.023520);0.9]〉,     〈[(0.005824,0.008400,0.011520);0.8],      [(0.004860,0.008400,0.013200);0.9]〉,     〈[(0.37,0.40,0.43);0.8],[(0.35,0.40,0.45);0.9]〉,     〈[(0.04,0.05,0.06);0.8],[(0.04,0.05,0.06);0.9]〉,     〈[(0.33,0.35,0.37);0.8],[(0.30,0.35,0.40);0.9]〉} =〈[(0.37,0.40,0.43);0.8],[(0.35,0.40,0.45);0.9]〉.

### 5.4. Comparisons and Discussion

**Figure 7:**
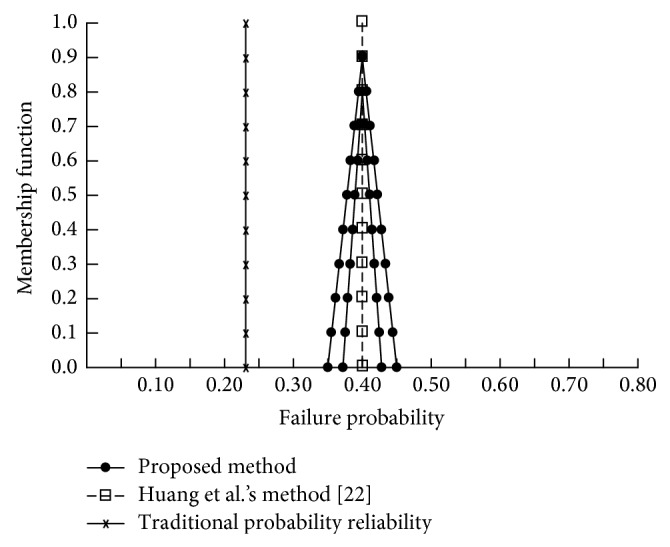
Membership function for top event of Internet security system failure.

In order to evaluate the proposed method, a numerical verification is performed in Section 5. This study also compares the experimental results with the traditional probability reliability and Huang et al.'s [22] methods. The input data of these methods are shown in Figure 5 and Table 1. In the comparison of the results of the three methods, the differences between the proposed method and the listing methods can be shown clearly in Figure 7. From Figure 7, there are some findings.

1. The traditional probability reliability and Huang et al.'s [22] methods do not consider the confidence level of domain experts. Therefore, the proposed method can be more flexible to present the confidence level of experts (highest confidence = 0.9).
2. In both the traditional probability reliability method and Huang et al.'s [22] methods, the failure possibilities of the top event are all equal to 0.2359 and 0.40. Because these methods are fit, the outcome of the top event is certain and precise as long as the assignment of basic events is decent from reliable information.
3. In the traditional probability reliability method, the failure possibilities of the top event are all equal at 0.2359. This is because the traditional probability reliability method does not consider the failure probability of the bottom events suffered in an attack and may obtain biased conclusions.
4. The results of the proposed and Huang et al.'s [22] methods under *α*-level 0.9 are the same.

From the comparison, it is clear that the integrated attack tree and vague set technique outlined in this study provides the following advantages. Firstly, the failure information is being described as vague variables; this results in a more realistic and flexible reflection of the real situation. Secondly, the proposed method has considered the failure probability of the bottom events suffered in the attack. Finally, the proposed approach can indeed help to solve security threat assessments of an Internet security system when the available information is incomplete.

## 6. Conclusions

This paper has proposed a novel technique to assess the security threats of an Internet security system while under attack. It is useful when evaluating system reliability using the available information and expert's expertise, which is often uncertain or vague in the Internet security system. In particular, this approach has considered the failure probability of the bottom events of an Internet security system suffered in an attack.

In order to further illustrate the proposed method and compare it with other techniques of traditional reliability methods, the Internet security system example is adopted as a simulation example. This study also compares the simulation results with the traditional probability reliability and Huang et al.'s [22] methods. The results show that the proposed approach could provide a more accurate and reasonable security threat assessment to assist the decision-making process. Furthermore, the presented approach is more realistic and is a flexible reflection of the real situation. Moreover, the proposed methodology can help engineers solve security threat assessment problems under the situation of vague or incomplete information.

The advantages of the proposed approach are summarized as follows.

1. The proposed method considers the malfunction data of the system elementary event as incomplete.
2. The proposed method provides more accurate and effective information to assist the decision-making process.
3. The failure information in a system's elementary event is described as vague variables; this result is more realistic and is a flexible reflection of the real situation.
4. From a hacker's point of view, finding the weak links in the system for design is to find out a better design of an Internet security system.

## Figures and Tables

**Table 1:** The possible range of leaf node failures.

Failure possibility	*a*_*i*_	*a*_*i*_′	*b*_*i*_	*c*_*i*_′	*c*_*i*_	μ_*A*_(*U*)	1 − ν_*A*_(*U*)
*A* (IP Table configuration errors)	0.30	0.33	0.35	0.37	0.40	0.8	0.9
*B* (address translation failure)	0.10	0.12	0.15	0.18	0.20	0.9	1.0
*C* (authentication failure)	0.80	0.90	0.10	0.11	0.12	0.9	0.9
*D* (Firewall Daemon configuration errors)	0.04	0.04	0.05	0.06	0.06	0.8	0.9
*E*1 (scan failure)	0.11	0.12	0.13	0.14	0.15	0.9	1.0
*E*2 (cleanse failure)	0.10	0.10	0.11	0.12	0.12	0.9	1.0
*E*3 (audit failure)	0.03	0.04	0.05	0.06	0.07	0.9	1.0
*E*4 (validation failure)	0.03	0.03	0.04	0.05	0.05	0.9	1.0
*F* (DNS configuration errors)	0.08	0.09	0.10	0.11	0.12	0.8	0.9
*G* (monitor service failure)	0.04	0.04	0.05	0.06	0.06	0.9	1.0
*H* (malicious access detected)	0.35	0.37	0.40	0.43	0.45	0.8	1.0
*I* (Transport Layer Security configuration errors)	0.12	0.13	0.14	0.15	0.16	0.8	0.9
*J* (peer entity authentication)	0.15	0.16	0.20	0.24	0.25	0.8	1.0
*K* (security parameter negotiation)	0.27	0.28	0.30	0.32	0.33	0.8	0.9
*L* (POP3 configuration errors)	0.16	0.17	0.18	0.19	0.20	0.8	0.9
*M* (entity authentication)	0.22	0.24	0.25	0.26	0.28	0.8	1.0
*N* (entry security parameter)	0.38	0.39	0.40	0.41	0.42	0.8	0.9
*O* (security parameter authentication)	0.07	0.08	0.09	0.10	0.11	0.8	1.0
*P* (key generation)	0.05	0.06	0.07	0.08	0.09	0.8	0.9
*Q* (data confidentiality)	0.03	0.03	0.04	0.05	0.05	0.9	0.9
